# A miniaturized 3D printed pressure regulator (µPR) for microfluidic cell culture applications

**DOI:** 10.1038/s41598-022-15087-9

**Published:** 2022-06-24

**Authors:** Meng-Chun Hsu, Mehran Mansouri, Nuzhet N. N. Ahamed, Stephen M. Larson, Indranil M. Joshi, Adeel Ahmed, David A. Borkholder, Vinay V. Abhyankar

**Affiliations:** 1 Department of Electrical Engineering, Rochester Institute of Technology, Rochester, NY 14623 USA; 2 Department of Biomedical Engineering, Rochester Institute of Technology, Rochester, NY 14623 USA

**Keywords:** Biomedical engineering, Mechanical engineering, Fluidics

## Abstract

Well-defined fluid flows are the hallmark feature of microfluidic culture systems and enable precise control over biophysical and biochemical cues at the cellular scale. Microfluidic flow control is generally achieved using displacement-based (e.g., syringe or peristaltic pumps) or pressure-controlled techniques that provide numerous perfusion options, including constant, ramped, and pulsed flows. However, it can be challenging to integrate these large form-factor devices and accompanying peripherals into incubators or other confined environments. In addition, microfluidic culture studies are primarily carried out under constant perfusion conditions and more complex flow capabilities are often unused. Thus, there is a need for a simplified flow control platform that provides standard perfusion capabilities and can be easily integrated into incubated environments. To this end, we introduce a tunable, 3D printed micro pressure regulator (µPR) and show that it can provide robust flow control capabilities when combined with a battery-powered miniature air pump to support microfluidic applications. We detail the design and fabrication of the µPR and: (i) demonstrate a tunable outlet pressure range relevant for microfluidic applications (1–10 kPa), (ii) highlight dynamic control capabilities in a microfluidic network, (iii) and maintain human umbilical vein endothelial cells (HUVECs) in a multi-compartment culture device under continuous perfusion conditions. We anticipate that our 3D printed fabrication approach and open-access designs will enable customized µPRs that can support a broad range of microfluidic applications.

## Introduction

Microfluidic approaches leverage the precise manipulation of fluids to introduce unique experimental capabilities in biological applications^1–3^, including the defined biophysical stimulation of cultured cells^4–8^, the controlled influx of chemical compounds^9–11^, and the introduction of secondary cell populations to the culture environment^12,13^. In these systems, control over fluid flow is typically achieved via displacement-based or pneumatic pumping schemes^14–16^. For example, syringe pumps use the rotary motion of mechanical screws to dispense fluid from a syringe barrel at a controlled flow rate (Q), while peristaltic pumps employ a cam mechanism to push or pull fluids through compliant tubing to directly control Q^17^. Although syringe and peristaltic pumps are frequently used because of their robust flow control capabilities and compatibility with standardized components (e.g., syringes, fittings, and tubing), they can be challenging to integrate into confined environments^18^. In addition, the mechanical oscillations of the screw or cam mechanism can introduce undesired flow pulsations that result in cell damage^19–22^.

In contrast, pneumatic pumping schemes create a defined pressure drop (ΔP) across microfluidic networks to control Q. For these pressure-driven flows, Q is defined by the Hagen–Poiseuille equation, Q = ΔPR^−1^, that can be thought of as the hydraulic analogy to Ohm’s Law, where R is the fluidic resistance defined by the geometry of the network and the fluid viscosity^23^. Because of the intrinsic damping nature of pneumatic systems, these approaches are less susceptible to flow pulsations compared to displacement-based methods^18^. However, because of potential fluidic resistance changes and concomitant back-pressure effects, pneumatic approaches often require complex peripheral equipment, such as a dedicated high-pressure air source (e.g., laboratory air), a closed-loop pressure controller, back-pressure regulators, and in-line pressure/flow sensors to maintain a desired flow rate^24–26^. Consequently, pneumatic methods can also be difficult to integrate into confined cell culture environments^27^.

Displacement and pneumatic techniques both offer excellent flow control capabilities and can be programmed to dynamically adjust flow profiles, including ramped, periodic, pulsed, or even reversed flows. However, these advanced features are often unused in standard microfluidic applications where a constant flow rate is used to perfuse or shear stimulate cultured cells^28,29^. The experimental popularity of constant, controlled perfusion rate allows us to prioritize a simple and portable pumping solution over one with advanced flow functionalities and complex instrumentation. Alternative approaches to simplify the pumping process have also been widely explored. For example, a commercial palm-top refillable iPrecio infusion pump was used to maintain cells in culture^30^. However, the pump was expensive, one-time use, and could not be customized. Alternatively, passive pumping, including hydrostatic and surface tension-based methods, are low-cost and easy to use but lack long-term stability, making them unsuitable for microfluidic culturing applications (> 24 h)^31–33^. Microelectromechanical systems (MEMS) approaches have also been used to create microfabricated pumps^34,35^. Although these micropumps can provide the long-term control required for lab-on-chip applications, the complexity of the fabrication procedures can make customization and implementation impractical.

3D printing, an emerging additive manufacturing technology, has been adopted as a fabrication method for highly customized microfluidic flow control devices because of rapid prototyping capabilities, low access costs compared to multi-axis CNC milling, and low tooling costs compared to injection molding^36–39^. 3D printing simplifies fabrication processes for features that are difficult to create (e.g., undercuts, freestanding features, and high aspect ratio cavities) using conventional machining technologies or MEMS processes. Researchers have successfully created 3D printed components for back pressure regulators^40^, Quake-style microvalves^41^, and pneumatic flow driving devices^26,42–44^.

To address the need for a simple but functional pumping platform, we introduce a pneumatic pumping platform that uses a 3D printed micro pressure regulator (µPR) to provide a tunable ∆P and control the flow rate in a microfluidic channel network. Our µPR uses a force-balance mechanism to reduce the pressure supplied by a battery-powered air pump to a controllable pressure range relevant to microfluidic applications. In this work, we detail the design and fabrication of the µPR, establish dynamic pressure control and stability characteristics, and demonstrate successful culture within a membrane-based, compartmentalized microfluidic barrier model^45,46^. As 3D printers have become broadly accessible in research laboratories and community makerspaces^47,48^, we anticipate that our 3D printed µPR—with open access designs—can be fabricated and assembled in any laboratory and tailored to achieve application-specific flow requirements.

## Materials and methods

### Fabrication of the pressure regulator

The structural components of the µPR, including the inlet (high-pressure) and outlet (low-pressure) chambers and the pressure control component, were 3D printed using the Formlabs Form 2 stereolithography printer (Formlabs Inc., Somerville, MA, USA). Dental SG resin (Formlabs Inc., Somerville, MA, USA) was selected as the building material due to its gas-impermeable characteristics and Class I biocompatibility (EN-ISO 10993-1:2009/AC:2010). The 3D printed parts were removed from the print platform, rinsed in 99% isopropyl alcohol, dried under pressurized air, and UV-cured for 45 min at 45 °C (FormCure, Formlabs Inc., Somerville, MA, USA), in accordance with the manufacturer’s recommendations.

**Figure 1:**
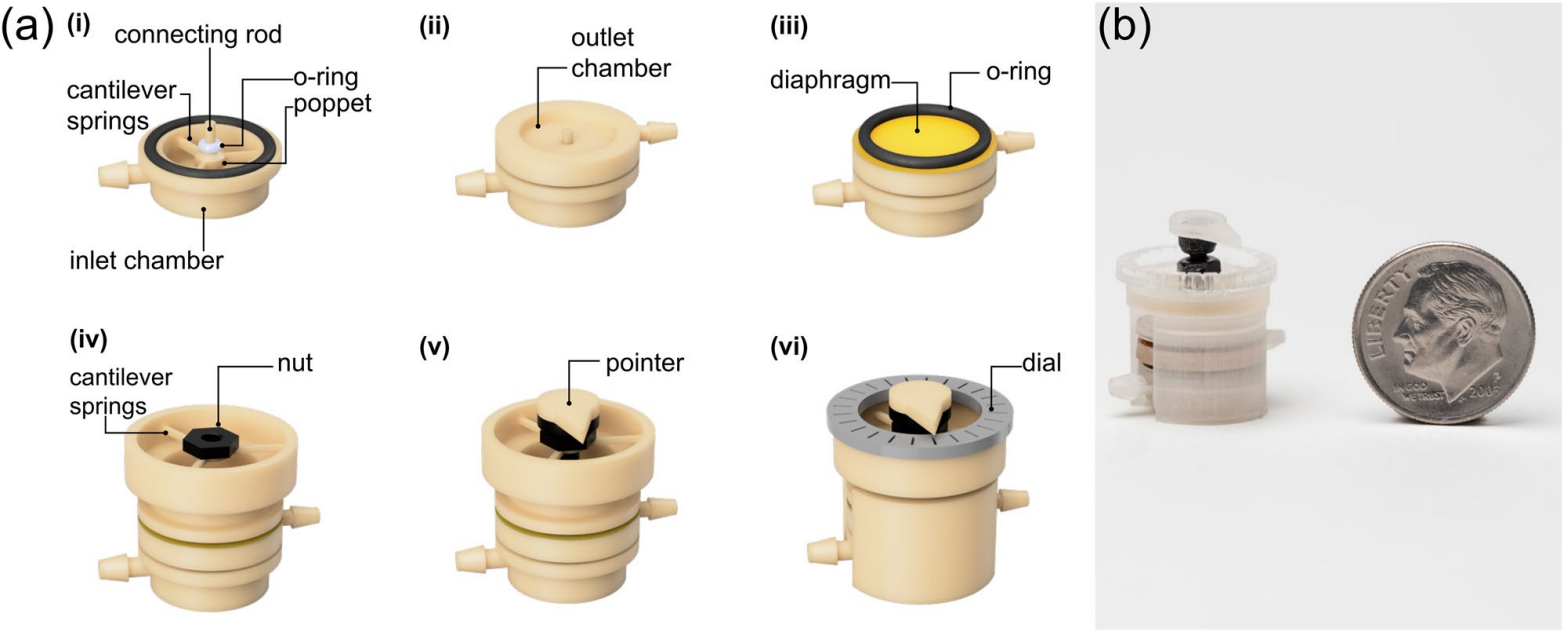
(**a**) Schematic view of the 3D printed µPR and fabrication workflow. (i) The high-pressure air inlet chamber includes a poppet valve, a sealing O-ring (white), and a connecting rod. (ii) The low-pressure air chamber is placed on top of the inlet chamber. (iii) A Kapton diaphragm (yellow) and O-ring (black) are placed atop the outlet chamber. (iv) The pressure control component, consisting of three built-in cantilevers and a threaded nut, is positioned on top of the O-ring. (v) An M2 bolt with a 3D-printed position indication pointer is threaded into the nut. (vi) The device is then sealed using two 3D-printed compression clamps to achieve an air-tight assembly (Φ12 mm × 20 mm) and a laser-cut position dial is added. (**b**) Image of the assembled 3D-printed µPR next to a United States dime for scale.

A 001 size Viton fluoroelastomer (shore 60A) O-ring (McMaster Carr, Elmhurst, IL, USA) was fitted over the connecting rod adjacent to the poppet valve of the high-pressure inlet chamber, as shown in Fig. 1a(i). An 8-mm-ID/10-mm-OD natural rubber (shore 70A) O-ring (McMaster Carr, Elmhurst, IL, USA) was then placed in the outer groove of the inlet chamber. The low-pressure outlet chamber, shown in Fig. 1a(ii), was placed over the inlet chamber with the connecting rod extending through the cavity to form the cross-chamber air passage. Next, a 100-µm thick Kapton (Gizmo Dorks LLC, Temple City, CA, USA) was placed onto the outlet chamber as the pressure sensing diaphragm, in contact with the connecting rod. As shown in Fig. 1a(iii), an O-ring was placed on top of the diaphragm to help seal the top of the outlet chamber. The pressure control component with built-in cantilever springs was then stacked onto the diaphragm. These cantilevers were 0.5 mm wide, 0.5 mm thick, and 5 mm long. An M2 nut (McMaster Carr, Elmhurst, IL, USA) was glued to the cantilever springs with epoxy adhesive (ClearWeld™ Professional, J-B Weld Company, Sulphur Springs, Texas, USA) (Fig. 1a(iv)). As shown in Fig. 1(v), an M2 bolt was threaded into the nut. A 3D printed pointer was added to the hexagonal socket head to create the control knob. A laser-cut, 24-position acrylic dial was attached to the pressure control component using pressure-sensitive adhesive (PSA, 3M 468MP Adhesive Transfer Tape, 3M Company, Maplewood, MN, USA). The dial provided indications for rotational positions in 15˚ increments. Finally, 3D printed clamps were used to compress the outer O-rings sandwiched between the structural components and complete the assembly, as shown in Fig. 1a(vi). The assembled device is 12 mm in diameter and 20 mm in height. Figure 1b shows an image of the assembled device next to a US dime for scale. See Supplemental Video S1 that shows the assembly process.

### Microfluidic channel fabrication

(Poly)dimethylsiloxane (PDMS, Sylgard 184, Dow Inc., Midland, MI, USA) microchannels were fabricated using standard soft lithography techniques^49,50^. SU-8 2100 (Kayaku Advanced Materials, Westborough, MA, USA) was spin-coated onto a 4″ silicon wafer, soft-baked, exposed to UV light through a transparency mask (CAD/Arts Services Inc., Bandon, OR, USA) to define channel features, and post-baked at 95 °C. The photoresist was then developed (Kayaku Advanced Materials, Westborough, MA, USA). A rectangular PMMA frame with open regions with length = 75 mm and width = 25 mm was attached to the wafer using PSA to create a molding cavity with a defined height. After attachment of the PMMA frame, the mold was then filled with degassed PDMS pre-polymer (10:1 base to catalyst ratio by mass) and cured on a hotplate for 1 h at 80 °C. The PDMS block was then removed from the mold and access ports were cored with a 1-mm biopsy punch (World Precision Instruments, Sarasota, FL, USA).

### COMSOL flow simulation setup

A 3D simulation was performed using the laminar flow physics (stationary) module in COMSOL Multiphysics. Microchannel geometry (20-µm height, 100-µm width, and 32-cm length) was applied with the material set as water. We assigned pressures (P = 1–10 kPa) to the inlet of the microchannel geometry, while the outlet pressure was defined as atmospheric (P = 0) with suppressed backflow. The other sides of the block were assigned no-slip boundary conditions.

### Pressure and flow rate measurement

The general experimental setup featured a µPR and a PDMS microfluidic flow resistor (20-µm height, 100-µm width, and 32-cm length). We supplied pressure to the µPR with a miniature DC air pump SX-2 (Binaca Pumps, Temecula, CA, USA) operating at 3 V and 0.09A. The outlet of the µPR was connected to a three-way connector, with one end feeding the inlet of the PDMS microfluidic channel and the other connected to a Honeywell pressure sensor (TBPDANS005PGUCV, Honeywell International Inc., Charlotte, NC, USA). Silicone tubing (2-mm ID, 5-cm length) was used to connect these components. The PDMS microchannel was primed with a solution of blue dye (McCormick Inc., Baltimore, MD, USA) in deionized water to improve contrast.

### Characterization of outlet pressure vs control knob position

The aforementioned experimental setup allowed characterization of P_out_ based on the angular position of the control knob. The control knob was turned by 15º increments (indicated with the acrylic dial) while P_out_ was monitored. P_out_ was then allowed to stabilize for 5-min at each position after turning the knob. A full cycle of the calibration process included clockwise rotational turns (P_out_ increased from 1 to 10 kPa) and counter-clockwise turns (P_out_ decreased from 10 to 1 kPa). 15 full cycles were used to calibrate the outlet pressure readings versus knob position. In order to quantify the stability of the regulated pressures, data was collected over a period of 1000 min for three designated pressures (P_out_ = 1, 5, and 10 kPa), covering the low, medium, and high set points of the range. It is important to note that to prevent the need for new calibration for different experimental setups, we use a large fluidic resistor (20 µm × 100 µm × 32 cm) after the pressure regulator. The fluidic resistor offered a much larger resistance than the other downstream elements and thus, allowed us to remove the flow sensors after the initial calibration step without requiring recalibration. This approach is equivalent to using a high input impedance to a maintain voltage drop in an electrical system.

### Maintenance of HUVECs in a membrane-based microfluidic platform using the µPR

Detailed design and fabrication of the barrier platform has been described in our previous work^45,46^. Briefly, the cell culture platform consisted of the top and bottom microchannels, separated by an ultrathin nanomembrane (SiMPore Inc., Rochester, NY, USA). The nanomembrane has a thickness of 100 nm and a pore size of 60 nm. The device has a core open-well module known as the m-µSiM which can be reconfigured into a fluidic device by adding a flow module into its well and sealing it magnetically using two housings with embedded magnets. The flow module was fabricated using standard soft lithography method and housings were fabricated using a laser cutter (H-series 20 × 12, Full Spectrum, CA, USA). The dimensions of the top channel were, h = 200 µm, w = 1.5 mm, and l = 5 mm, and the bottom channel were, h = 150 µm, w = 2–6 mm, and l = 15 mm. The flow from the media reservoir was connected to the inlet of the top channel using tubing and 21 gauge 21 NT dispensing tips (Jensen Global, USA).

Before use, the flow circuit was sterilized via exposure to UV light^46^. Since the air pump was used inside an incubated and sterile environment, further filtration of the output air was not required. Prior to cell seeding, the nanomembrane was coated with 5 µg cm^−2^ fibronectin (Corning Inc., Corning, NY, USA) for one hour at room temperature, and then rinsed with fresh cell media. Human umbilical vein endothelial cells (HUVECs) (Thermo Fisher Scientific, Waltham, MA, USA) were cultured in EBM-2 Basal Medium (Lonza Bioscience, Walkersville, MD, USA) supplemented with EGM-2 Endothelial Cell Growth Medium-2 BulletKit (Lonza Bioscience, Walkersville, MD, USA) and maintained in a tissue culture flask. Prior to use, cells were dissociated using TrypLE (Thermo Fisher Scientific, USA) for 3 min and centrifuged at 150 G for 5 min. After re-suspension, cells were seeded onto the membrane surface through the top microchannel and incubated for 1 h to promote cell attachment.

The µPR was set to an output pressure of 8 kPa (∆P = 8 kPa), which corresponded to a media flow rate of 1 µL min^−1^ (shear stress of 0.02 dynes cm^−2^ at cell monolayer) in the top channel for 24 h. LIVE/DEAD stain (Thermo Fisher Scientific, Waltham, MA, USA) was used to assess cell viability based on the vendor’s protocol. Labelled cells were imaged using an Olympus IX-81 fluorescence microscope with CellSens software (Olympus, Tokyo, Japan) with constant image capture settings across the experimental sets.

### Dynamic control visualization using laminar flow streams

A Y-shaped PDMS microchannel consisting of two 1-cm-long inlet channels and a 1-cm-long outlet channel was connected to two µPRs (P1 and P2) and two battery-powered micropumps. Each µPR was connected to a pressure sensor to measure pressure. P1 was maintained at 1.0 kPa while P2 was varied. We allowed 30 s for each P2 stage to provide a sequence of pressures: 1.0 kPa, 1.3 kPa, 1.0 kPa, 1.5 kPa, 1.0 kPa, 1.8 kPa, and 1.0 kPa, for a total of 3 min and 30 s. The liquid–liquid interface between colored streams was recorded with an SMZ-168 stereomicroscope and its camera (Motic Co., ltd., Xiamen, China). Data are reported as mean value ± standard deviation.

## Results

### A force-balance mechanism enables a tunable range of outlet pressures

**Figure 2:**
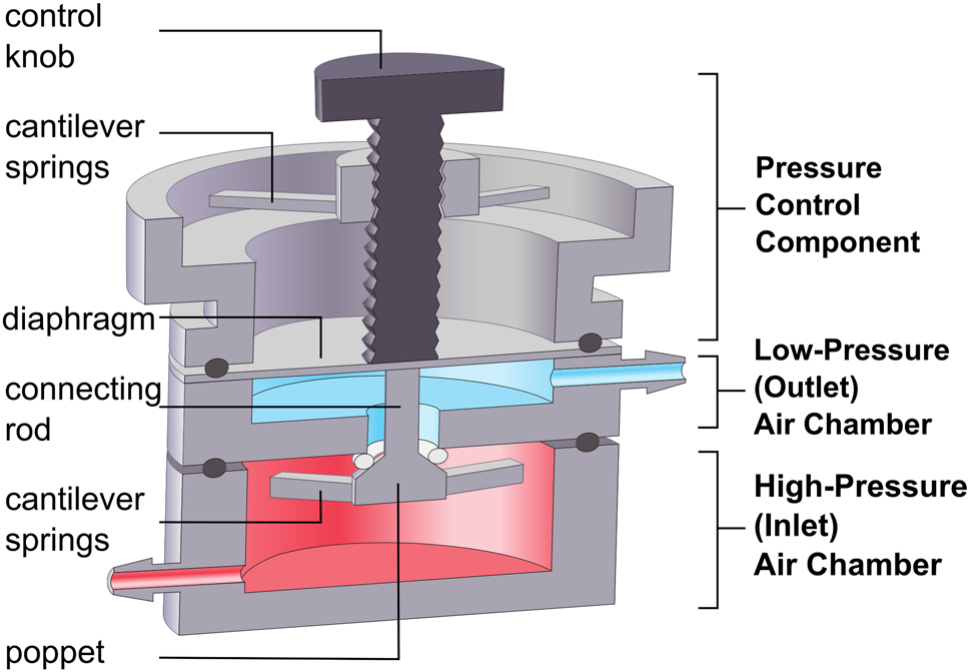
Cross-sectional schematic of essential components in the 3D-printed µPR. The high-pressure chamber (red) receives a constant high-pressure air supply from an external source. The low-pressure chamber (blue) outputs air at a constant low-pressure value. The outlet pressure is controlled by adjusting the pressure control component, consisting of cantilever springs and a control knob.

**Figure 3:**
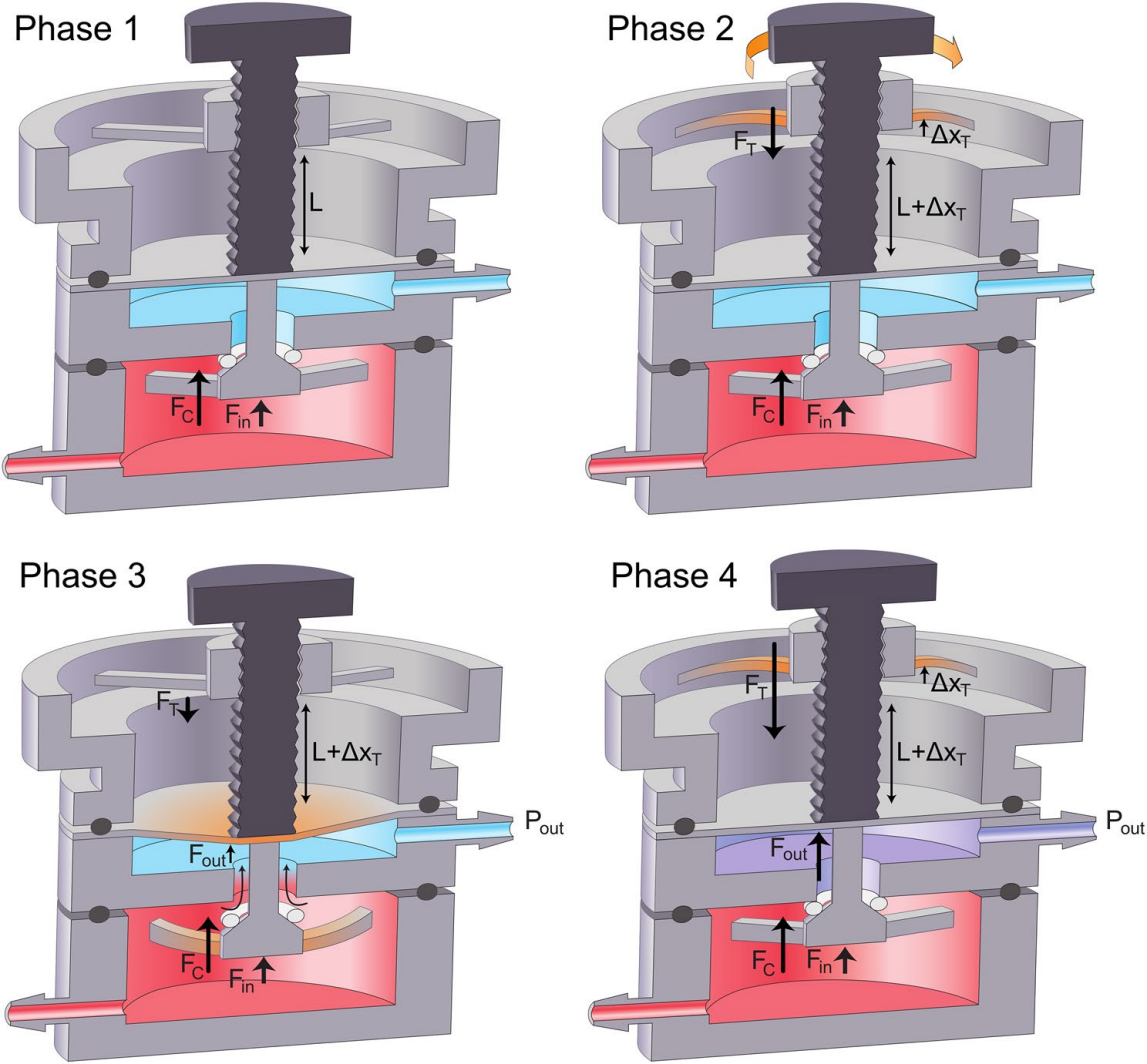
Depiction of the four phases of the pressure regulating process. During Phase 1, the air passage is fully closed, while we supply air from a constant high pressure source. In Phase 2, the user turns the control knob to displace the top cantilevers. As the top cantilever restoring force (F_T_) increases, the air passage between the chambers remains closed. In Phase 3, when F_T_ surpasses a threshold value, the air passage opens. Finally, in Phase 4, the pressure in the low-pressure air chamber reaches the desired level set by the position of control knob and the passage will close. Once the pressure is set by the user, the device cycles between Phase 3 and Phase 4 to maintain the desired output pressure.

Pressure regulators are commonly used in pneumatic circuits to reduce high-pressure air to a lower, controllable pressure setpoint for downstream applications. As with most manual pressure regulators, our 3D printed µPR uses a force-balance mechanism and is designed to maintain a user-defined setpoint suitable for standard microfluidic systems (~ 1–10 kPa). As shown in Fig. 2, the µPR consists of a high-pressure air chamber, low-pressure air chamber, and pressure control component. The high-pressure air chamber includes the closing (bottom) cantilever springs, the poppet valve, and the connecting rod. This chamber receives constant pressure from a miniature air pump. The low-pressure chamber with the pressure sensing diaphragm outputs the regulated outlet pressure. The pressure control component consists of 3D printed top cantilever springs and the control knob (a bolt and a pairing nut), which is used to control the outlet pressure as described below. The operation of µPR can be described in four phases as shown in Fig. 3.

#### Phase 1

Constant high-pressure air is supplied to the high-pressure chamber using a miniature air pump. There are two closing forces present at this stage. The inlet pressure force (F_in_) is an upward force generated by the inlet pressure acting on the poppet. The closing cantilever spring force (F_C_) is a constant upward force generated by the displacement of the non-adjustable bottom cantilever springs and is set during assembly. These upward forces press the poppet to the seat and close the air passage between chambers. In this phase, the bolt length under the nut is L and the tip of the bolt rests against the pressure sensing diaphragm without exerting a downward force.

#### Phase 2

As we turn the control knob clockwise, the bolt length under the nut is increased to (L + ΔX_T_) and the top cantilever springs are displaced upward from their relaxed state by (ΔX_T_). This upward displacement of the cantilever springs generates a downward restoring force (F_T_ = k_T_ΔX_T_) on the sensing diaphragm. During this phase, the air passage is still sealed by upward forces (F_in_ and F_C_) because F_T_ < F_in_ + F_C_.

#### Phase 3

When the control knob is rotated further to increase ΔX_T_, F_T_ overcomes the upward forces (F_in_ + F_C_) and the bolt tip displaces the pressure sensing diaphragm and connecting rod downward. The motion of the connecting rod unseats the poppet valve and opens the air passage, allowing high-pressure air to enter the low-pressure chamber. The pressure (P_out_) in the low-pressure chamber exerts an upward force (F_out_) on the bottom surface of the pressure sensing diaphragm (area A_d_), P_out_ = F_out_ A_d_^−1^.

#### Phase 4

P_out_ increases until the summation of F_out_ and other upward forces F_in_, F_C_ is equal to F_T_ as shown in Eq. (1). These upward forces push the poppet valve toward the seat and block air flow between chambers (Fig. 3). This allows P_out_ to be set by changing the top cantilever spring force (F_T_ = k_T_ ΔX_T_) by adjusting the rotational position of the control knob. Since P_out_ is used to pressurize a downstream fluid reservoir or channel P_out_ decreases and the µPR re-enters Phase 3 to allow high-pressure air to compensate for the pressure loss. Once ΔX_T_ is set by the control knob, the µPR cycles between Phases 3 and 4 to maintain a stable setpoint, P_out_.

1 $${F}_{T}={F}_{out}+{F}_{in}+{F}_{C}$$

Here, the top cantilever spring force F_T_ = k_T_ΔX_T_; k_T_ is the spring constant of the top cantilever spring and ΔX_T_ is the spring displacement. The outlet pressure force F_out_ = P_out_A_d_; P_out_ is the outlet pressure and A_d_ is the area of the sensing diaphragm. F_in_ is the inlet pressure force on the exposed area of the poppet, and F_C_ is a constant closing force from the bottom cantilever springs.

Equation (1) simplifies because the closing cantilever springs in the high-pressure chamber are not adjustable, and therefore F_C_ is a constant. F_in_ is constant as long as we supply a constant input pressure to the high-pressure chamber. Because both F_in_ and F_C_ are constants, we can control F_out_ (thus P_out_) by manipulating the F_T_ applied to the diaphragm. F_T_ scales linearly with the displacement (ΔX_T_) of the top cantilever springs, hence we can tune P_out_ by adjusting the angular position of the control knob. Supplemental Video S2 shows an animation of the pressure regulation process.

### Calibration of the µPR and characterization of output pressure stability

**Figure 4:**
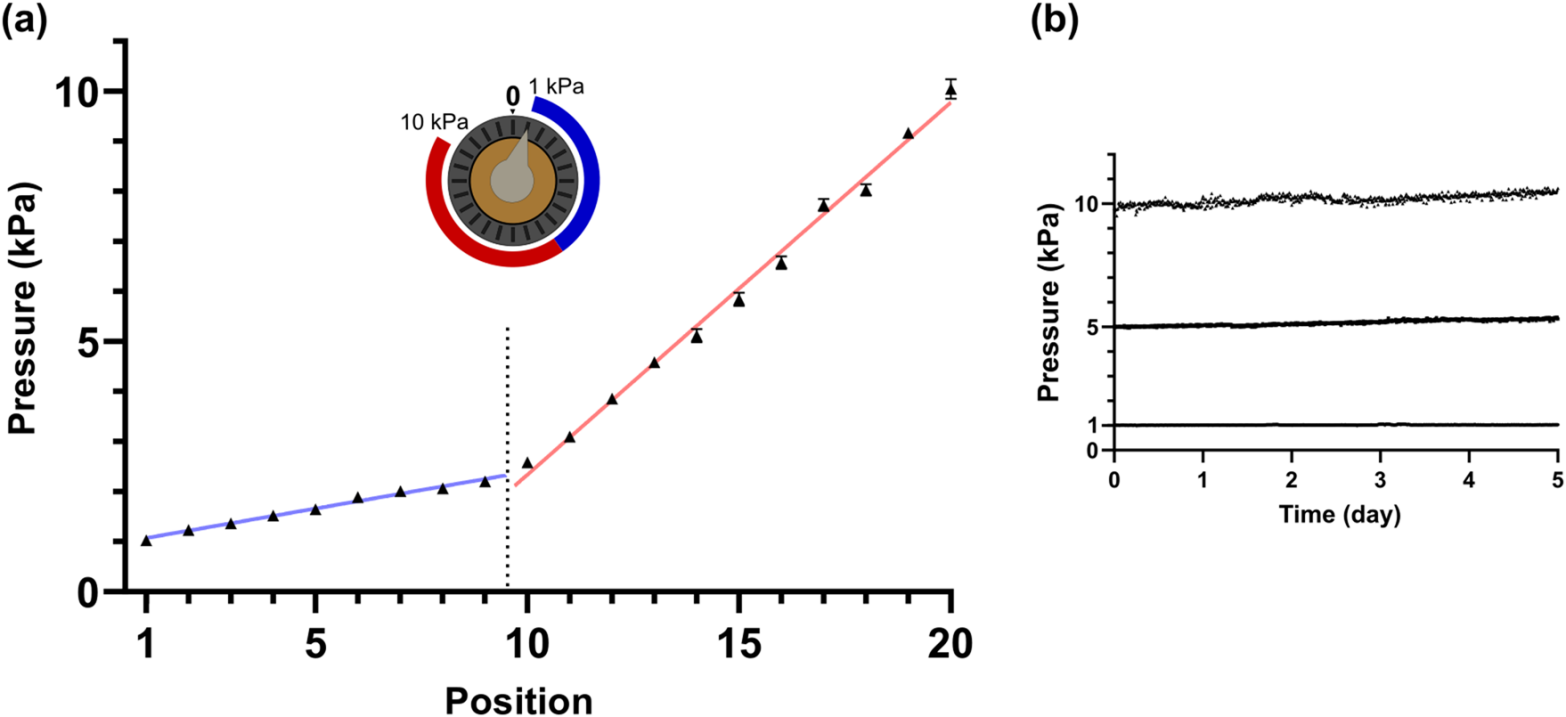
(**a**) Outlet pressure vs. control knob positions (15º steps). Pressures increased by 0.15-kPa increments between the 1st and 9th positions (blue), and 0.70-kPa increments between the 10 and 20th positions (red). (**b**) Outlet pressure stability test with pressures set to 1, 5, and 10 kPa, by turning the control knob to the 1st, 14th, and 20th positions, respectively, following the calibrated results in (**a**). The pressure was measured over 5 days to check the stability of the outlet pressure regulated by the device. The three outlet pressures were 1.1 ± 0.01 kPa, 5.2 ± 0.11 kPa, and 10.2 ± 0.20 kPa throughout the 5-day stability test.

A major goal of our pumping platform is to provide tunable pressure control while maintaining a portable setup. Therefore, we selected a miniature battery-powered air pump instead of a compressed air line or a pressurized cylinder as the external high-pressure source. Since our µPR operates on the assumption of constant inlet pressure (see Eq. (1)), we first confirmed that the pressure from the miniature air pump was stable over time. Running at 3 V, the pump maintained a stable pressure (41 ± 0.02 kPa) over the course of 5 days*.* Next, we sought to characterize the relationship between the angular position of the control knob and the resulting outlet pressure. As shown in Fig. 4a, we rotated the control knob by 15˚ increments (corresponding to increasing or decreasing ΔX_T_ in Fig. 3) and measured the output pressure. The data revealed two distinct slopes. In the first region from 1st to the 9th position (1.0–2.2 kPa), the slope was 0.15 kPa per 15° increment while in the second region from the 10th to 20th position (2.6–10 kPa) the slope was 0.70 kPa per 15° increment. These different slopes may be a consequence of the compressibility of the sealing O-ring on the poppet valve. That is, the O-ring may be partially in contact with the valve seat and limiting air flow between chambers (positions 1 to 9). With increased rotation (positions 10 to 20), the O-ring detaches fully from the valve seat and air can flow between chambers with less resistance, thus creating a steeper slope relationship.

To ensure controlled flow for culture applications, it is important to provide a stable pressure drop (∆P = P_out_ − P_atm_) across the microchannel network. Here, the outlet pressure (P_out_) regulated by the µPR helps establish $\Delta P$. Using the calibration data from Fig. 4a, we characterized the stability of P_out_ over 5 days at three different setpoints, 1, 5, and 10 kPa. As shown in Fig. 4b, the outlet pressures were 1.1 ± 0.01 kPa (1.1% error), 5.2 ± 0.11 kPa (2.2% error), and 10.2 ± 0.20 kPa (1.9% error) and demonstrated the µPR’s ability to provide tunable and stable pressures across the output range.

**Figure 5:**
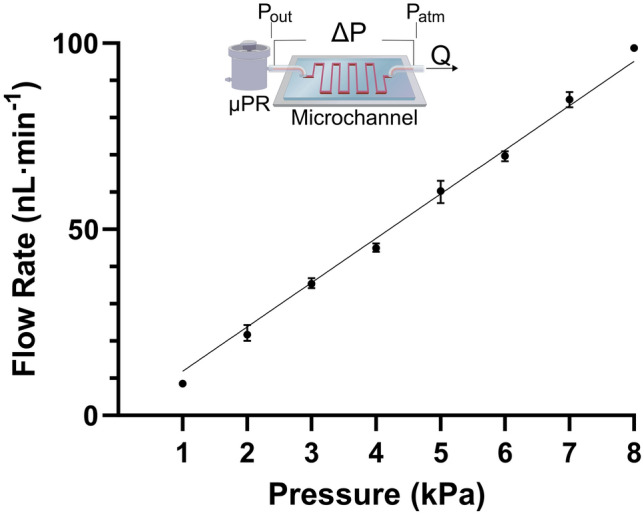
The inset shows the test setup, including the pressure regulator that creates a ∆P across the microchannel. ∆P is determined by the outlet pressure of µPR and the atmospheric pressure at the end of the microchannel. ∆P (1 to 8 kPa) covers flow rates from 8.50 to 98.7 nL min^−1^, with the relationship described by the slope 12 nL min^−1^ kPa^−1^. The straight line is the simulated response of flow rates vs. the outlet gauge pressures. R^2^ = 0.999 is the correlation between the experimental data and the COMSOL simulation results.

Next, we explored how the µPR could be used to provide a stable pressure drop across a microfluidic channel and produce flow rates practical for cell culture applications. The µPR was designed to support low flow rates that can be difficult to achieve with commercial pressure regulators (e.g., 10–100 nL min^−1^). The flow rates were measured in Fig. 5 for different outlet pressures to quantify the µPR’s capability of controlling the liquid flow. We introduced pressure drops, ∆P, from 1 to 8 kPa, using the µPR and measured flow rates ranging from 8.50 to 98.7 nL min^−1^. We observed an excellent correlation (R^2^ = 0.999) between the COMSOL simulations and experimental flow rate measurements (∆P from 1 to 8 kPa). The slope describing the relationship is 12 nL min^−1^ kPa^−1^.

### Human umbilical vein endothelial cells (HUVECs) culture in a multi-compartment microfluidic platform

**Figure 6:**
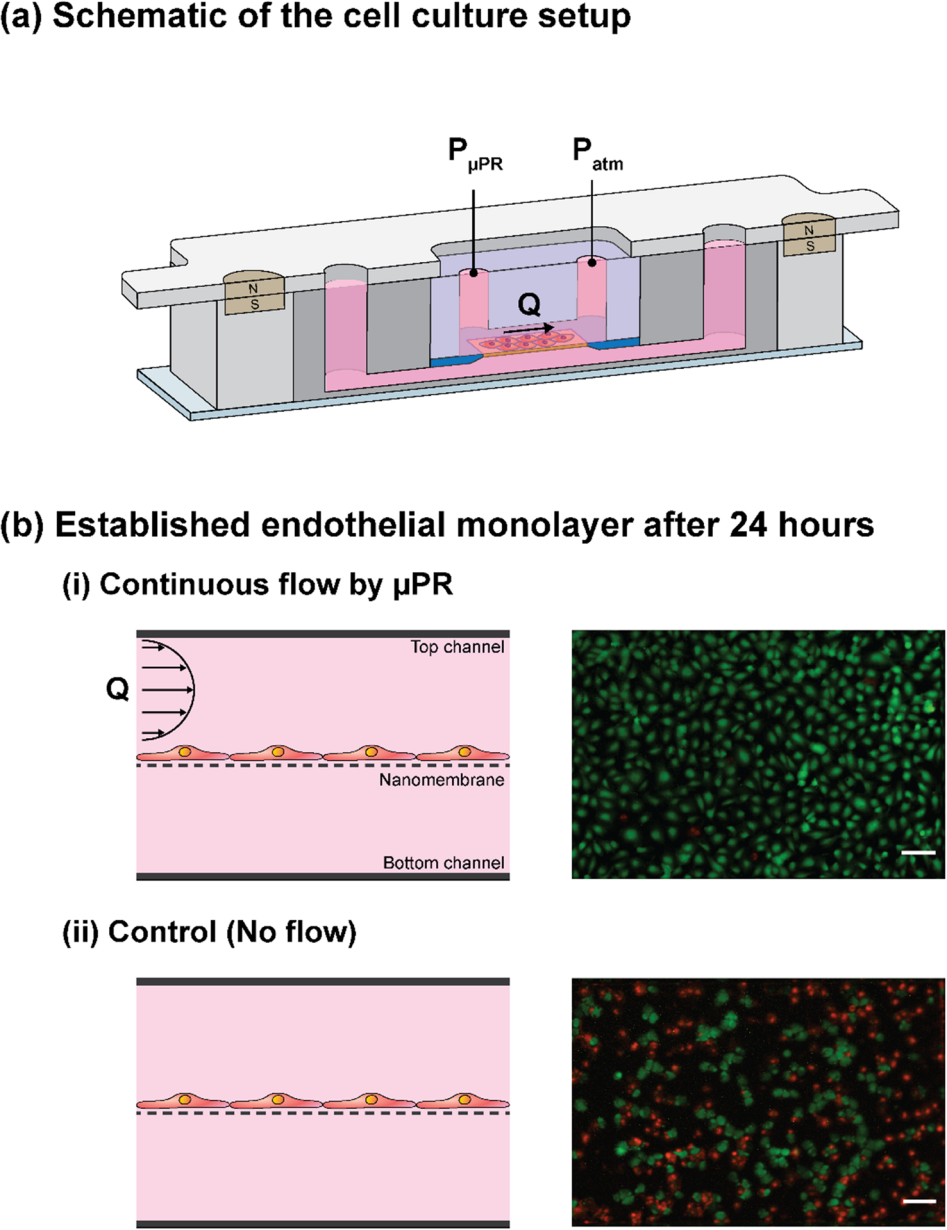
(**a**) Schematic illustration of the cell culture platform. A mini air pump supplies high-pressure air to µPR, which outputs a stable pressure drop (ΔP) across the top microchannel of the platform. This results in the flow of cell media from the reservoir into the microchannel. The platform consists of two microchannels separated by an ultrathin nanomembrane. Components of the platform can be disassembled after the experiment due to its reversible magnetic latching mechanism. We set the output of 8 kPa from the µPR to drive the culture media flow (Q = 1 µL min^−1^). (**b**) Cross-sectional view of the endothelial monolayer, and comparison of cultured cells in (i) dynamic culture (with the flow) and (ii) static culture (no flow). The cells were stained with LIVE/DEAD stain and fluorescence images were captured in green (viable cells) and red (dead cells). This demonstrates that the µPR can drive continuous flow vital for long-term cell culture and the formation of a confluent cell monolayer. Scale bars = 100 µm.

In microfluidic systems, media perfusion is required because the microliter-scale media volume in the channel is rapidly depleted of nutrients by metabolically active cells and must be replenished to maintain cell viability. To demonstrate the compatibility of our µPR to control fluid flow and maintain cells, we used the µPR to establish an endothelial monolayer in a tissue barrier model that we previously developed^46^. As shown in Fig. 6a, the culture platform consists of two microchannels separated by a nanomembrane. The lower channel was filled with cell media while the top channel was supplied with flows driven by the µPR. The µPR induced a stable pressure drop of 8 kPa across the top culture microchannel, resulting in a constant 1 µL min^−1^ flow rate for introducing cell media from the reservoir into the culture region.

As expected, cells cultured in the device with media flow driven by the µPR were maintained alive and formed a confluent monolayer after 24 h while the majority of cells in the static control died due to lack of cell media supply (Fig. 6b). The live/dead staining showed 98% survival rate in the µPR-supplied device whereas the static control (no media flow) had a 38% survival rate. These results confirmed the capability of the µPR to deliver stable flow rates and maintain a long-term culture of cells in microfluidic devices.

### Outlet pressure can be dynamically changed by adjusting the control knob position

**Figure 7:**
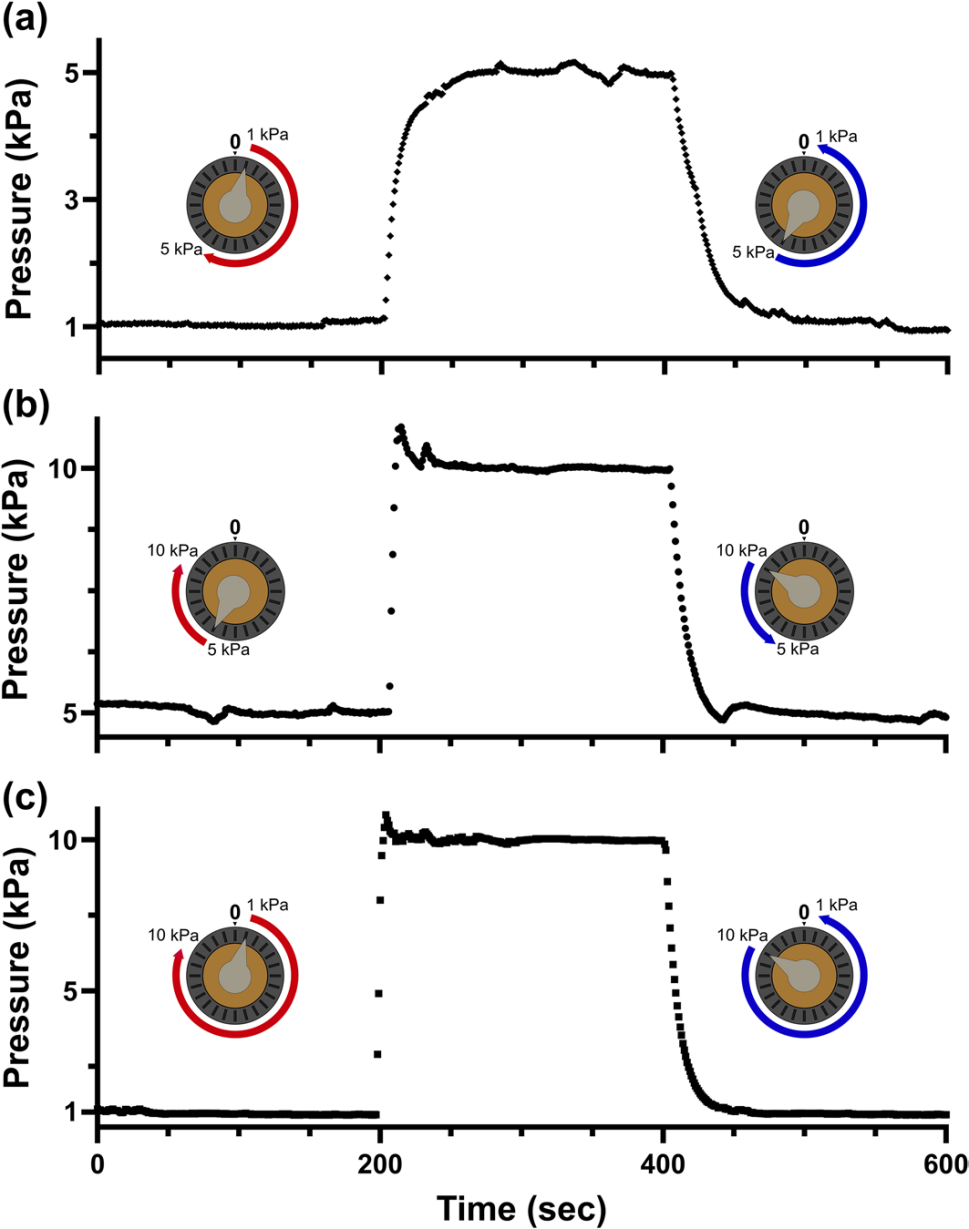
Dynamic responses of pressure patterns including, (**a**) 1 to 5 to 1 kPa, (**b**) 5 to 10 to 5 kPa, and (**c**) 1 to 10 to 1 kPa are achieved via control knob turns with the calibration data in Fig. 4a. Each pattern features three stages, each with 200 s under the real-time observation of the dynamic pressure response.

Since the outlet pressure can be easily changed based on the calibrated position of the control knob, we demonstrate µPR’s responsiveness to real-time pressure switching. As shown in Fig. 7, we show dynamic step changes in pressure that spanned the entire pressure range: (a) 1 kPa–5 kPa–1 kPa, (b) 5 kPa–10 kPa–5 kPa, and (c) 1 kPa–10 kPa–1 kPa. In this experiment, we again used the calibration results as presented in Fig. 4a for setpoints of the control knob positions for pressures used in this experiment. Figure 7 shows that our µPR could ramp up and down to reach desired setpoints within one-minute periods, even among the largest dynamic pressure patterns in the experiment.

To highlight the integration of multiple µPRs in a single system, we utilized two µPRs to separately control the flow rates of two liquids within a Y-shaped microfluidic channel and visualized the dynamic equilibrium position of the dual-stream laminar flow interface while adjusting one µPR to a new setpoint. We fed red-dyed deionized water to the top inlet port of the Y-channel with the pressure set to 1.0 kPa µPR, P1. Blue-dyed deionized water was fed into the bottom inlet port with pressure regulated by a second µPR, P2; these pressure values were changed during the experiment from a range of 1.0 kPa to 1.8 kPa.

**Figure 8:**
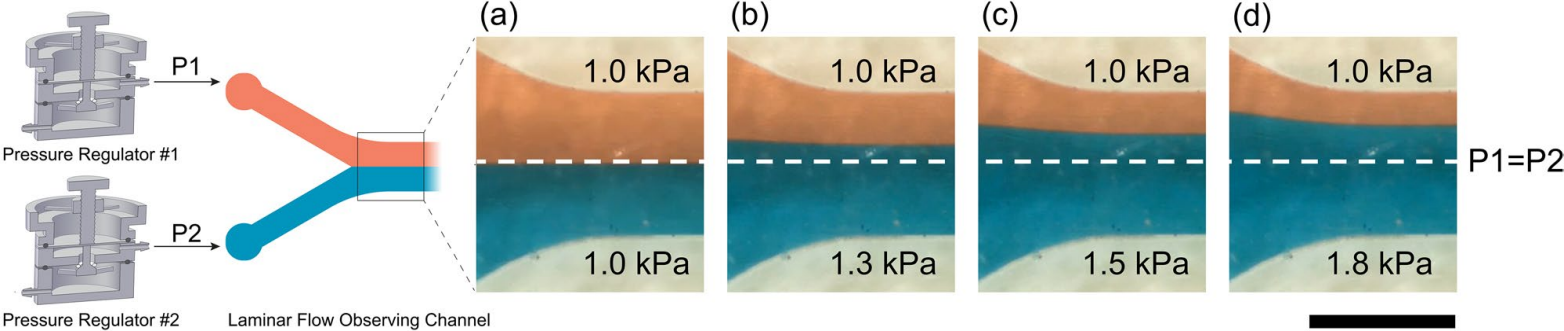
Real-time observation of co-laminar liquid flow pressurized with two µPRs. µPR #1 supplies pressure (P1) to one inlet port of the laminar flow observing channel, while µPR #2 supplied pressure (P2) to the other. P1 was set to 1 kPa, while P2 was adjusted to (**a**) 1.0 kPa, (**b**) 1.3 kPa, (**c**) 1.5 kPa, and (**d**) 1.8 kPa using the control knob. Scale bar = 1 mm.

As expected, when P1 = P2, the liquid–liquid interface between the red and blue streams was located at the midline of the channel (white dashed line), confirming the capability of delivering stable flow rates using multiple µPRs. As we changed P2 from 1.0 to 1.8 kPa by turning the control knob, the flow rate in the bottom channel increased and the interface was shifted upward (see Fig. 8 and Supplemental Video S3, shown at 8x speed). We allowed a 30-s period of observation time for each new P2 set point with the following sequence of pressures: 1.0 kPa, 1.3 kPa, 1.0 kPa, 1.5 kPa, 1.0 kPa, 1.8 kPa, and 1.0 kPa, for a total of 3 min and 30 s. The liquid–liquid interface shifted in response to the P2 pressure adjustment, quickly settled to the new position, and maintained stability during each of the 30-s pressure monitoring periods. The dynamic response of the µPR flow adjustment demonstrated real-time pressure adjustment and stable dynamic equilibrium positions. We highlighted the pressure control capabilities of the system and flow profile possibilities for more advanced real-time features that require pressure controls.

## Discussion

The goal of our platform is to provide a portable, simplified microfluidic flow control method while providing stable flows suitable for cell culture applications. While there are commercial solutions for pneumatic pressure control, these pressure regulators have larger footprints (> 30 mm), a higher outlet pressure range (~ 35 kPa) with a lower resolution (> 3.5 kPa). These approaches also cannot be customized, are expensive (> $100 USD for one with aforementioned features), and require a dedicated laboratory compressed air line. These techniques are summarized in Table S2. By introducing the µPR along with a mini air pump to create a microfluidic flow control platform, we can deliver a range of tunable and stable flow rates within a portable system. Our platform provides a cost-effective pressure control scheme with a range of customization opportunities owing to the increasing availability of hobby and commercial 3D printers. For reference, the total cost of the mini air pump and µPR setup as shown in this work is less than $7 USD, of which the µPR is less than $1.20 as shown in supplementary Table S1.

In our design, (see Figs. 2 and 3), the pressure regulating mechanism is similar to that of conventional pressure regulators. However, by incorporating 3D printing techniques, we were able to integrate two sets of cantilever springs as an alternative to large commercial springs to simplify the assembly and help miniaturize the device. By incorporating cantilever springs into the poppet valve design, we created an upward closing force (F_C_), as shown in Fig. 3, to prevent possible high-pressure air leakage to the low-pressure chamber through the air passage. This “normally-closed” design allows users to shut off output pressure and momentarily disconnect the cell culture compartments for inspection or modification. Since regulation of P_out_ depends on the closing actions of the poppet valve, we chose a gas-impermeable elastomeric O-rings (shore 60A) at the poppet for better sealing. This suits our target applications, which are often operated with a low-pressure and low flow rate regime. To target the range of 1–10 kPa, we chose the M2 size (0.4-mm pitch, 2-mm diameter) bolt as the control knob with a 24-position dial. Such a combination provides sufficient pressure resolution (< 1 kPa per 15° turn) while retaining user-friendly control. By adjusting some key mechanical parameters, such as k_T_ and A_d_ we can achieve different targeted outlet pressure ranges. Equation (2) shows that k_T_ can be modified by changing the mechanical properties of the cantilever by either switching to a different material or changing the curing settings of the 3D printer. k_T_ can also be altered by the geometry of cantilevers. For example, we can increase the pressure sensitivity when we decrease k_T_—which can be achieved by increasing the length of the cantilever springs or decreasing their width or thickness, as shown in Eq. (2).

2 $${k}_{T}=\frac{3Eb{h}^{3}}{4{l}^{3}}$$

where E is the Young’s modulus of the 3D printed material, and b, h, l are the width, thickness, and length of each cantilever, respectively.

Although k_T_ is more sensitive to changes in the thickness (h) of the cantilever than the width (b) (see Eq. (2)), the z accuracy (i.e., layer thickness control) of the 3D printer is often less than the x–y axes, resulting in greater variability in the thickness^51^. For example, a 0.1-mm thickness change (from 0.5 to 0.6 mm) of the cantilever springs can result in 70% increase in the spring constant. We anticipate that the 3D printed µPR can be modified to fit different pressure ranges based on the mathematical descriptions. For example, increasing the area of the sensing diaphragm A_d_ can improve the resolution of the output pressure setpoint but results in a larger device footprint and a smaller upper bound (constrained by the maximum cantilever spring force) of the outlet pressure, since the outlet force scales linearly with the diaphragm area but is limited by the top cantilever spring force.

We fabricated our device by stacking 3D printed components with an O-ring as a key sealing component to separate the high and low air pressure chambers. Multi-material 3D printers can be used to print this device in one fabrication step with rigid and flexible parts for robust sealing, but these printers may not be accessible in all laboratories. For single-material printers, print-pause-print techniques could allow placement of soft materials for sealing during the fabrication but would add fabrication complexity and uncertainty^52^. Since 3D printed structures are still associated with dimensional errors for such small device features, each device needs to be calibrated to determine the relationship between knob position and output pressure with the mathematical equations serving as general design guidelines. The relationship between control knob positions and outlet pressures, once calibrated, can be used to produce the desired outlet pressure in other applications. The µPR does not come into contact with fluid and can be reused as needed. The compact and easy setup of the µPR-based microfluidic flow control platform provides manual control of ΔP based on the calibration. Since the flow control platform depends on the pressure drop to achieve the required flow rates, we used an open-ended system (pressure at channel outlet = P_atm_) to limit back pressure effects. During the cell culture experiment, we were able to deliver a constant flow rate of media to the culture platform to maintain a viable environment for HUVECs as compared to the no-flow situation. For more complicated microfluidic networks or closed-end systems, users can add back-pressure regulators to raise the downstream pressure threshold at the end of the microfluidic network to prevent potential backflows, however, this would require a higher range of driving pressures to deliver the same flow rates^26,53^.

With the dynamic control capability demonstrated with the co-laminar flows, we present more possibilities in dynamically controlling the outlet pressure to introduce different media flow rates for culture setup changes using our µPR (e.g., shear stress adjustment for cell alignment purposes) without modifying the channel geometry. In contrast to syringe pumps and commercial pneumatic solutions, the small footprint and minimal peripheral equipment requirements of the µPR-based system and be easily moved in and out of a cell culture incubator. Although this work is focused on creating an ease-of-use, tunable, and simple cell culture platform for single constant flow rates, automated flow control functionalities including pulsed or controlled ramp flows could be introduced using a stepper motor and gear train to program adjustments to the knob positions.

## Conclusions

In summary, we introduce an easy-to-fabricate, low-cost, miniaturized 3D printed µPRs and highlighted stable pressure control capabilities relevant to many microfluidic applications. We also demonstrated that the µPR and pumping platform could be used to maintain cells in a compartmentalized, membrane-based microfluidic culture environment. We anticipate that our simple fabrication techniques and open-access design files will enable other laboratories to customize µPRs to support a broad range of microfluidic applications where syringe pumps or traditional pneumatic methods are challenging or inconvenient to integrate.

## Supplementary Information

Supplementary Information 1.

Supplementary Video S1.

Supplementary Video S2.

Supplementary Video S3.

## Data Availability

All data is available upon reasonable request. The pressure regulator CAD design files are available from https://abhyankarlab.org.
