# Supplementary material for: A miniaturized 3D printed pressure regulator (µPR) for microfluidic cell culture applications
Source: Sci Rep. 2022 Jun 24;12:10769. doi: 10.1038/s41598-022-15087-9 (PMC9232624; doi:10.1038/s41598-022-15087-9)
Supplement: Supplementary file 1 — Supplementary Information 1. [file 41598_2022_15087_MOESM1_ESM.pdf]

A Miniaturized 3D-Printed Pressure Regulator (μPR) for Microfluidic Cell Culture Applications

Meng-Chun Hsu[1,2], Mehran Mansouri[2], Nuzhet N.N. Ahamed[2], Stephen M. Larson[2], Indranil M. Joshi[2], Adeel Ahmed[2], David A. Borkholder[1], and Vinay V. Abhyankar[2*]
Departments of Electrical Engineering[1] and Biomedical Engineering[2]
Rochester Institute of Technology, Rochester, NY 14623

Supporting Information

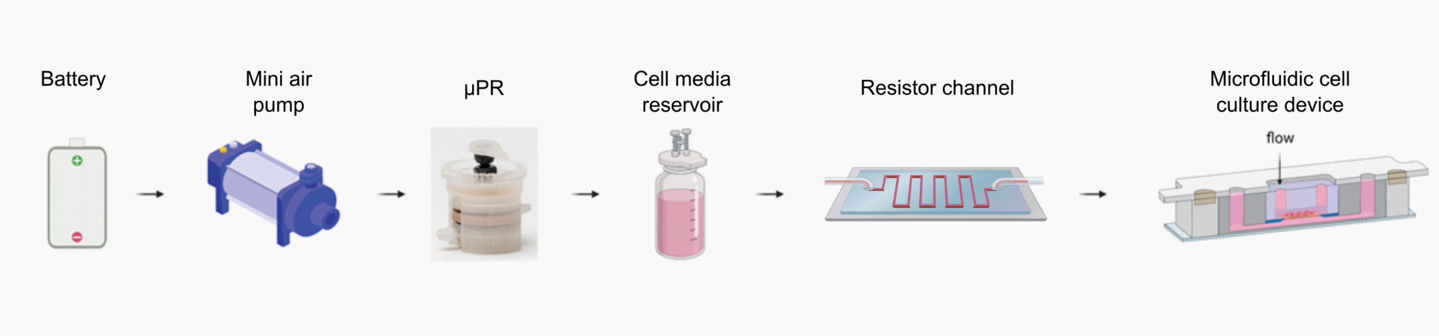

Fig. S1. Schematic of the cell culture process workflow. The AA batteries were used to power the mini air pump, which produced a high pressure that was regulated by the μPR. The μPR pressurized the sealed cell media reservoir to provide necessary media flow to the microfluidic cell culture device.

Table. S1. Bill of materials for the μPR and mini air pump set up

Item	Stock Number	Quantity	Unit price	Adjusted Price
Dental SG Resin	FLDGOR01	1.24 mL	$299/Liter	$0.37
Viton O-ring (001)	1284N101	1	$5.80/(25 pieces)	$0.23
Oil-resistant Buna-N O-ring	9262K442	2	$17.67/(100 pieces)	$0.35
M2 nut	91828A111	1	$6.14/(100 pieces)	$0.06
M2 bolt	91290A012	1	$16.58/(100 pieces)	$0.17
**μPR total**				**$1.18**
**Mini air pump**		**1**	**$10.99/(2 pieces)**	**$5.50**
**Total**				**$6.68**

Table. S2. Benchmark table of compressed-air based flow driving set ups

Regulation Method	Description	Cost	Size (mm)	Pressure Accuracy (kPa)	Input/Output Pressures (kPa)	Stablity	Compressed Air Line / Power Supply	Commercial Product	Reference
Diffusion	3D-printed casing components were used to make a pressure regulator that can reduce 1-2 bars of pressures by 0-1 bars. Pressure regulating is dictated by the permeability of PDMS membranes.	Not reported	Φ35 x 24	Not reported	100-200 / 0-200	Not reported	Yes / No	No	Podwin 2018[1]
Electromagnetic	A miniaturized pressure regulating valve to lower pressure (glaucoma range) by employing a combination of solenoid/permanent magnet. Fluidic driving was not the major purpose of this work.	Not reported	9.2 x 9.2 x 3.1	Not reported	2.7-4 / 2.7	Not reported	No / Yes	No	Bae 2003[2]
Electromagnetic	Automated pneumatic setup with solenoid valves, pressure regulators, and relief needle valves. The marginal costs of increasing streams are lower than syringe pump networks.	> $700	Network of pressure components	3.5	280 / 3.5-28	Not reported	Yes / Yes	No	Bong 2011[3]
Electromagnetic	The electromagnetically-controlled device regulated input pressure by applying different current to the coil (71Ω). The switching frequency of the valve can be up to 30 Hz. The device can operate at inlet pressure up to 200 kPa (with 300mA current).	Not reported	10 x 10 x 4.3	Not reported	50-200 / 0-125	Not reported	Yes / Yes	No	Fu 2003[4]
Electromagnetic	Kudasik utilized a solenoid valve, a pressurized cylinder, and a buffering cylinder to stabilize required pressures.	Not reported	Network of pressure components	0.1 kPa	1400-2000 / 100-700	0.1 kPa over 18h	Yes / Yes	No	Kudasik 2010[5]

Table. S2. Benchmark table of compressed-air based flow driving set ups (cont'd)

Regulation Method	Description	Cost	Size (mm)	Pressure Accuracy (kPa)	Input/Output Pressures (kPa)	Stablity	Compressed Air Line / Power Supply	Commercial Product	Reference
Electromagnetic	Liu et al. presented control methods, including Bang-Bang, proportional and pulse-width-modulation, and composite control, to electromagnetically control pressures in a micro chamber.	Not reported	3 x 3 x 1 (chamber)	3.15	220-280 / 150	3.15	Yes / Yes	No	Liu 2017[6]
Electrostatic	Anjewierden reported a parylene-based electrostatic device that can operate with pressures up to 40 kPa. Flow rate average recorded at 1.05 mL/min. The flow rate is also dependent on parylene membrane's thickness.	Not reported	20 x 75 x (30, estimated)	Not reported	0-40 / 0-40	Not reported	Yes / Yes	No	Anjewierden 2012[7]
Electrostatic	Yıldırım created an electrostatically-operated normally-closed parylene microvalve that can withstand up to 20 kPa. The actuation chamber for the valve has a radius of 410μm.	Not reported	Not reported	Not reported	0-20 / 0-20	Not reported	Yes / Yes	No	Yıldırım 2012[8]
Electrostatic	Yoshida created an electrostatic microvalve to regulate inlet pressure up to 40 kPa without leakage. By manipulating the duty ratio of the microvalve, Yoshida was able to deliver desired flow rates of methanol.	Not reported	5.5 x 12.5 x 2.3	Not reported	0-40 / 0-40	Dependent on duty ratio	Yes / Yes	No	Yoshida 2010[9]
Mechanical	Begolo 3D-printed a device with a pumping lid that creates the differential pressure for driving fluids in microchannels. The device was printed with multi-material 3D printer with sealing elastomers.	Not reported	14.73 cm^3	2.5	NA / < 20	30% over 7 hrs	No / No	No	Begolo 2014[10]

Mechanical	Beswick Engineering provides a commercially available pressure regulator specifically for low pressure applications. The website offers material options for different components of the pressure regulator.	$100	Φ26 x 50	3.5	< 3500 / < 350	0.1% full scale	Yes / No	Yes	Beswick Engineering[11]
Mechanical	Mavrogiannis introduced system with one inlet and four outlets for microfluidics applications. The flow rates are dictated by the incorporated commercial pressure regulators and fluidic resistances of coupled capillaries. We calculated the stability based on flow fluctuations.	$500	Network of pressure components	3.5	< 350 / < 100	6.70%	Yes / No	No	Mavrogiannis 2016[12]
**Mechanical**	**We utilized a mini air pump to supply a pressure to be regulated by our 3D-printed *μ*PR to create an affordable device for flow control. Cost of *μ*PR alone is $1.19.**	**$6.68**	**Φ12 x 20**	**0.7**	**60 / 1-10**	**2% set point**	**No / Yes**	**No**	**Current manuscript**
Mechanical	Thrugood introduced an affordable flow delivery system for microfluidics using a latex balloon reinforced by nylon stockings. The balloon can be manually squeezed to reach desired pressures.	$2	Φ250	Manual	2.5-25 / 2.5-25	9.4% over 9h	No / No	No	Thurgood 2019[13]
Not reported	A USB-powered low-pressure pump up to 50 kPa. The device is compatible with LabVIEW can be coupled with control valves for multi-inlet switching.	$2,500	100 x 60 x 30	Not reported	NA / 0-50	Not reported	No / Yes	Yes	Dolomite Mitos Fluika Pump[14]
Piezoelectric	The device comes with pressure and vaccuum inlets can be coupled with flow sensors for driving flows in both directions.	>$20,000	240 x 223 x 80	0.001	150-1000 / 0-20	0.001kPa	Yes / Yes	Yes	Elveflow Systems[15]

Piezoelectric	Evans created a feed-back controlled system with a spring-pressurized reservoir (37mL) as air tank, a piezoelectric microvalve, and a flow sensor to deliver desired flow rates.	Not reported	45 x 85 x 34	Not reported	0-15 / 0-15	3.22% Flow	No / Yes	No	Evans 2010[16]
Piezoelectric	Nafea utilized a pressurized balloon (up to 13 kPa) and a normally-closed piezoeolectric microvalve to deliver flows. The device is wirelessly powered by an inductor-capacitor circuit at 10 kHz.	Not reported	22 x 42 x 4	Not reported	13 / Not reported	Not reported	No / Yes	No	Nafea 2018[17]

Thermo-pneumatic	Chee utilized an external coil to heat an air reservoir locally to obtain the pressure required to drive flows. The maximum pressure obtained in the device was 0.4 kPa with frequency at 81.6 kHz. Variations in pumping strokes were documented at 2.8%	Not reported	22 x 7 x 4	Not reported	NA / 0-50	2.80%	No / Yes	No	Chee 2015[18]
Thermo-pneumatic	Cooney powered a pump with a coil locally heating the reservoir. The flow rates required a warm-up stage, where a 18% deviation was reported. The device, powered by an alkaline 9V battery, could deliver 1.4 uL/min for at least 4.5 hours.	Not reported	Φ20 x 1 (pump only)	Not reported	NA / 7-42	18%	No / Yes	No	Cooney 2004[19]
Thermo-pneumatic	Henning created a mass flow controller/pressure regulator by combining a normally-open and a normally-closed valve. The device was intended to regulate ultra-low pressure.	Not reported	106 x 40 x 25	1% full scale	172-345 / 1.3	0.1% full scale	Yes / Yes	discontinued	Henning 1998[20]

1. Podwin, A., Walczak, R. & Dziuban, J. A 3D printed membrane-based gas microflow regulator for on-chip cell culture. *Appl. Sci.* **8**, (2018).

2. Bae, B. *et al. In vitro experiment of the pressure regulating valve for a glaucoma implant*. (2003).

3. Bong, K. W. *et al.* Compressed-air flow control system. *Lab Chip* **11**, 743–747 (2011).

4. Fu, C., Rummler, Z. & Schomburg, W. *Magnetically driven micro ball valves fabricated by multilayer adhesive film bonding INSTITUTE OF PHYSICS PUBLISHING JOURNAL OF MICROMECHANICS AND MICROENGINEERING Magnetically driven micro ball valves fabricated by multilayer adhesive film bonding*. *J. Micromech. Microeng* vol. 13 http://iopscience.iop.org/0960-1317/13/4/316 (2003).

5. Kudasik, M., Skoczylas, N., Sobczyk, J. & Topolnicki, J. Manostat - An accurate gas pressure regulator. *Meas. Sci. Technol.* **21**, (2010).

6. Liu, X. & Li, S. Control method experimental research of micro chamber air pressure via a novel electromagnetic microvalve. in *Proceedings - 2017 4th International Conference on Information Science and Control Engineering, ICISCE 2017* 921–925 (Institute of Electrical and Electronics Engineers Inc., 2017). doi:10.1109/ICISCE.2017.195.

7. Anjewierden, D., Liddiard, G. A. & Gale, B. K. An electrostatic microvalve for pneumatic control of microfluidic systems. *J. Micromechanics Microengineering* **22**, (2012).

8. Yıldırım, E., Arikan, M. A. S. & Külah, H. A normally closed electrostatic parylene microvalve for micro total analysis systems. *Sensors Actuators, A Phys.* **181**, 81–86 (2012).

9. Yoshida, K., Tanaka, S., Hagihara, Y., Tomonari, S. & Esashi, M. Normally closed electrostatic microvalve with pressure balance mechanism for portable fuel cell application. *Sensors Actuators, A Phys.* **157**, 290–298 (2010).

10. Begolo, S., Zhukov, D. V., Selck, D. A., Li, L. & Ismagilov, R. F. The pumping lid: Investigating multi-material 3D printing for equipment-free, programmable generation of positive and negative pressures for microfluidic applications. *Lab Chip* **14**, 4616–4628 (2014).

11. Beswick Engineering Pressure Regulator. https://www.beswick.com/catalog/product-category/regulators/.

12. Mavrogiannis, N., Ibo, M., Fu, X., Crivellari, F. & Gagnon, Z. Microfluidics made easy: A robust low-cost constant pressure flow controller for engineers and cell biologists. *Biomicrofluidics* **10**, (2016).

13. Thurgood, P. *et al.* Self-sufficient, low-cost microfluidic pumps utilising reinforced balloons. *Lab Chip* **19**, 2885–2896 (2019).

14. Dolomite Mitos Fluika Pump. https://www.dolomite-microfluidics.com/product/mitos-fluika-pump/.

15. Systems, E. Elveflow Microfluidic Flow Controller. https://www.elveflow.com/microfluidic-products/microfluidics-flow-control-systems/ob1-pressure-controller/.

16. Evans, A. T., Park, J. M., Chiravuri, S. & Gianchandani, Y. B. A low power, microvalve regulated architecture for drug delivery systems. *Biomed. Microdevices* **12**, 159–168 (2010).

17. Nafea, M., Nawabjan, A. & Mohamed Ali, M. S. A wirelessly-controlled piezoelectric microvalve for regulated drug delivery. *Sensors Actuators, A Phys.* **279**, 191–203 (2018).

18. Chee, P. S., Minjal, M. N., Leow, P. L. & Ali, M. S. M. Wireless powered thermo-pneumatic micropump using frequency-controlled heater. *Sensors Actuators, A Phys.* **233**, 1–8 (2015).

19. Cooney, C. G. & Towe, B. C. A thermopneumatic dispensing micropump. *Sensors Actuators, A Phys.* **116**, 519–524 (2004).

20. Henning, A. K. *et al.* Microfluidic MEMS for semiconductor processing. *IEEE Trans. Components Packag. Manuf. Technol. Part B* **21**, 329–336 (1998).
